# Network pharmacology insights into the mechanistic basis of Taohe Chengqi Decoction in the treatment of constipation

**DOI:** 10.1097/MD.0000000000047501

**Published:** 2026-02-06

**Authors:** Yulai Yin, Yixuan Xie, Shufa Tan, Yinxu Zhang, Chen Xu

**Affiliations:** a School of Medicine, Nankai University, Tianjin, China; b School of Intergrative Medicine, Tianjin University of Traditional Chinese Medicine, Tianjin, China; c Department of General Surgery, The First Affiliated Hospital of Jinzhou Medical University, Jinzhou, China; d Department of Colorectal Surgery, Tianjin Union Medical Center, The First Affiliated Hospital of Nankai University, Tianjin, China.

**Keywords:** AGE–RAGE signaling, constipation, network pharmacology, protein–protein interaction, Taohe Chengqi Decoction

## Abstract

Constipation is a common gastrointestinal disorder associated with impaired motility, inflammation, and altered neuro-intestinal regulation. Taohe Chengqi Decoction, a classical prescription from *Shang Han Lun*, has been widely applied in the treatment of constipation, yet its pharmacological mechanisms remain insufficiently understood. We integrated systems pharmacology and network analysis to elucidate the therapeutic mechanisms of Taohe Chengqi Decoction against constipation. Active compounds and their putative targets were retrieved from traditional Chinese medicine systems pharmacology and PubChem, while constipation-related genes were collected from GeneCards and OMIM. Shared targets were identified and subsequently analyzed using STRING to construct a protein–protein interaction network. Hub proteins were ranked by degree centrality. A drug–disease–target network was built to map the interactions between Taohe Chengqi Decoction and constipation. Gene ontology and Kyoto encyclopedia of genes and genomes enrichment analyses were performed to uncover functional modules and signaling pathways. A total of 188 common targets were identified. Protein–protein interaction network analysis highlighted AKT1, interleukin-6 (IL6), IL1B, and JUN as hub proteins, suggesting central roles in regulating inflammation, apoptosis, and signal transduction. Additional nodes with high connectivity, such as caspase-3, PTGS2, signal transducer and activator of transcription 3, hypoxia-inducible factor-1α, estrogen receptor 1, and epidermal growth factor receptor, were implicated in apoptosis, oxidative stress, and transcriptional regulation. The drug–disease–target network revealed a dense and highly interconnected structure, reflecting the multicomponent, multi-target nature of Taohe Chengqi Decoction. Kyoto encyclopedia of genes and genomes enrichment indicated significant involvement of the advanced glycation end-product binding to their receptor signaling pathway, along with IL-17, TNF, and HIF-1 pathways, underscoring the contribution of inflammatory and oxidative stress-related processes. This study, based on computational pharmacology analysis, predicts that Taohe Chengqi Decoction may exert therapeutic effects on constipation through an integrated regulation involving multiple components, targets, and pathways. The potential mechanisms are likely associated with the modulation of inflammatory responses, apoptosis, and oxidative stress, with the advanced glycation end-product binding to their receptor signaling pathway possibly acting as a key mediator. These findings provide theoretical insights and future directions for elucidating the molecular mechanisms underlying the therapeutic effects of Taohe Chengqi Decoction against constipation.

## 1. Introduction

Constipation^[1–3]^ is a prevalent clinical condition characterized by reduced bowel movement frequency (fewer than 3 per week), hard stools, difficult defecation, or a persistent sensation of incomplete evacuation. It is broadly classified into functional constipation (comprising slow-transit, outlet obstruction, and mixed types) organic constipation, which arises from structural abnormalities, metabolic disorders, neurological dysfunction, or adverse drug effects, and acute or chronic forms. The underlying mechanisms involve impaired colonic motility, diminished rectal sensitivity, pelvic floor dyssynergia, abnormal neural regulation, and alterations in fluid balance. Chronic constipation may lead to colonic dilatation, atrophy of the intestinal muscular layer, mucosal injury, and complications such as hemorrhoids, anal fissures, and intestinal obstruction.

Within the framework of traditional Chinese medicine (TCM),^[4]^ constipation of the excess-heat or blood stasis-heat type is frequently attributed to Yangming bowel excess (intestinal heat with fluid depletion) or qi stagnation with blood stasis (emotional disturbance impairing qi and blood flow). Taohes Chengqi Decoction, a representative formula recorded in the Shanghan Lun,^[5]^ is composed of Semen Persicae (Taoren), Radix et Rhizoma Rhei (Dahuang), Ramulus Cinnamomi (Guizhi), Radix Glycyrrhizae (Gancao), and Natrii Sulfas (Mangxiao). The formula exerts a synergistic action through promoting blood circulation and resolving stasis (Taoren), purgation and stool softening (Dahuang and Mangxiao), unblocking meridians (Guizhi), and harmonizing other components (Gancao). Together, these herbs act to dispel blood stasis, clear heat, and relieve constipation, making Taohes Chengqi Decoction particularly suitable for constipation associated with blood stasis and heat accumulation. Its putative mechanisms include stimulating intestinal motility, improving microcirculation, and modulating enteric nervous system function, thereby alleviating symptoms and reducing complications. Network pharmacology is a scientific approach that investigates drug–target–disease associations based on the integration of pharmacological targets and disease-related genes. Its applicability, however, is largely influenced by the selection and quality of databases. To mitigate potential bias arising from reliance on a single source, multiple databases were employed to identify disease-associated genes and thereby enhance the robustness of the analysis.

Although clinical studies have suggested the efficacy of Taohes Chengqi Decoction in constipation,^[6,7]^ the underlying pharmacological mechanisms remain poorly understood. To address this gap, the present study employs a network pharmacology approach to elucidate the mechanistic basis of Taohes Chengqi Decoction in the treatment of constipation.

## 2. Materials and methods

### 2.1. Screening of active components in Taohes Chengqi Decoction

The major constituents of Taohes Chengqi Decoction (Semen Persicae [Taoren], Radix et Rhizoma Rhei [Dahuang], Ramulus Cinnamomi [Guizhi], Radix Glycyrrhizae [Gancao], and Natrii Sulfas [Mangxiao]) were retrieved from the Traditional Chinese medicine systems pharmacology database and analysis platform (TCMSP, https://www.tcmsp-e.com).^[8]^ Candidate compounds were screened using oral bioavailability (OB) >30% and drug-likeness (DL) >0.18 as the thresholds. Since Mangxiao was not indexed in TCMSP, its active components were further queried in PubChem (https://pubchem.ncbi.nlm.nih.gov)^[9]^ using “mirabilite” as the search term. Corresponding target genes of the identified active compounds were annotated against the UniProt human gene database (https://www.uniprot.org/).^[10]^ The OB value in TCMSP is predicted using the OBioavail 1.1 model, which integrates support vector machine, multiple linear regression, and partial least squares algorithms to estimate the oral absorption efficiency of compounds. The DL value is calculated based on the Tanimoto similarity coefficient, which evaluates the structural similarity between each compound and known marketed drugs using molecular descriptors. Compounds with OB ≥30% are considered to possess favorable bioavailability and are likely to represent the bioactive constituents of traditional Chinese medicine formulas in vivo. According to the TCMSP database, the average DL value of approved drugs is approximately 0.18; therefore, a threshold of DL >0.18 was adopted to indicate compounds with physicochemical properties similar to typical drugs and with potential for further development.

### 2.2. Identification of constipation-related targets

Constipation-associated gene targets were extracted from GeneCards (https://www.genecards.org)^[11]^ and OMIM (https://www.omim.org)^[12]^ using “constipation” as the search keyword. The retrieved targets from both databases were merged after removing duplicates to obtain a comprehensive set of constipation-related genes. In this study, potential targets were collected from multiple databases, including TCMSP, GeneCards, and OMIM. To ensure data consistency, all target names were standardized by converting them to their official gene symbols using the UniProt database with *Homo sapiens* selected as the species. Duplicate targets were then removed, and only validated, unique targets were retained. This process ensured consistency and standardization of target information across databases, allowing for reliable data integration.

### 2.3. Identification of common targets and construction of the protein–protein interaction (PPI) network

Common targets between Taohes Chengqi Decoction and constipation were identified using the R package VennDiagram, and Venn diagrams were plotted accordingly. PPI networks of overlapping targets were generated via the STRING database (https://string-db.org) under the “Multiple proteins” setting, with the minimum interaction score set at the value of 0.700. Isolated nodes were retained. The resulting interaction networks and tab-separated values files were exported and subsequently analyzed in Cytoscape (version 3.9.0; The Cytoscape Consortium, San Diego) for topological parameters to identify core targets within the network. Specifically, in the intersection analysis between drug targets and disease-associated targets, we applied a hypergeometric distribution model to calculate the significance level of their overlap, thereby verifying the nonrandomness and statistical reliability of the shared targets. This test effectively assesses whether the observed overlap between drug and disease targets exceeds what would be expected by chance, thus enhancing the credibility of the subsequent PPI network analysis.

### 2.4. Gene ontology (GO) and Kyoto encyclopedia of genes and genomes (KEGG) enrichment analyses

GO and KEGG enrichment analyses were performed in R using the “colorspace,” “stringi,” “DOSE,” “clusterProfiler,” and “pathview” packages. To control the false discovery rate arising from multiple hypothesis testing in the network pharmacology analysis, the Benjamini–Hochberg correction was applied. Pathways with adjusted *P*-values <.05 were considered statistically significant. The top 20 enriched pathways, ranked by adjusted *P*-value, were visualized as bar plots. In addition, pathway diagrams were generated for signaling pathways exhibiting high inter-pathway correlations to facilitate further mechanistic interpretation.

## 3. Results

### 3.1. Identification of overlapping targets between Taohes Chengqi Decoction and constipation

Using the TCMSP and PubChem databases, a total of 140 active compounds were retrieved from Semen Persicae (Taoren), Radix et Rhizoma Rhei (Dahuang), Ramulus Cinnamomi (Guizhi), Radix Glycyrrhizae (Gancao), and Natrii Sulfas (Mangxiao). These compounds corresponded to 2745 predicted targets. After gene annotation and removal of duplicates, 209 unique drug-related genes were obtained.

**Figure 1.**
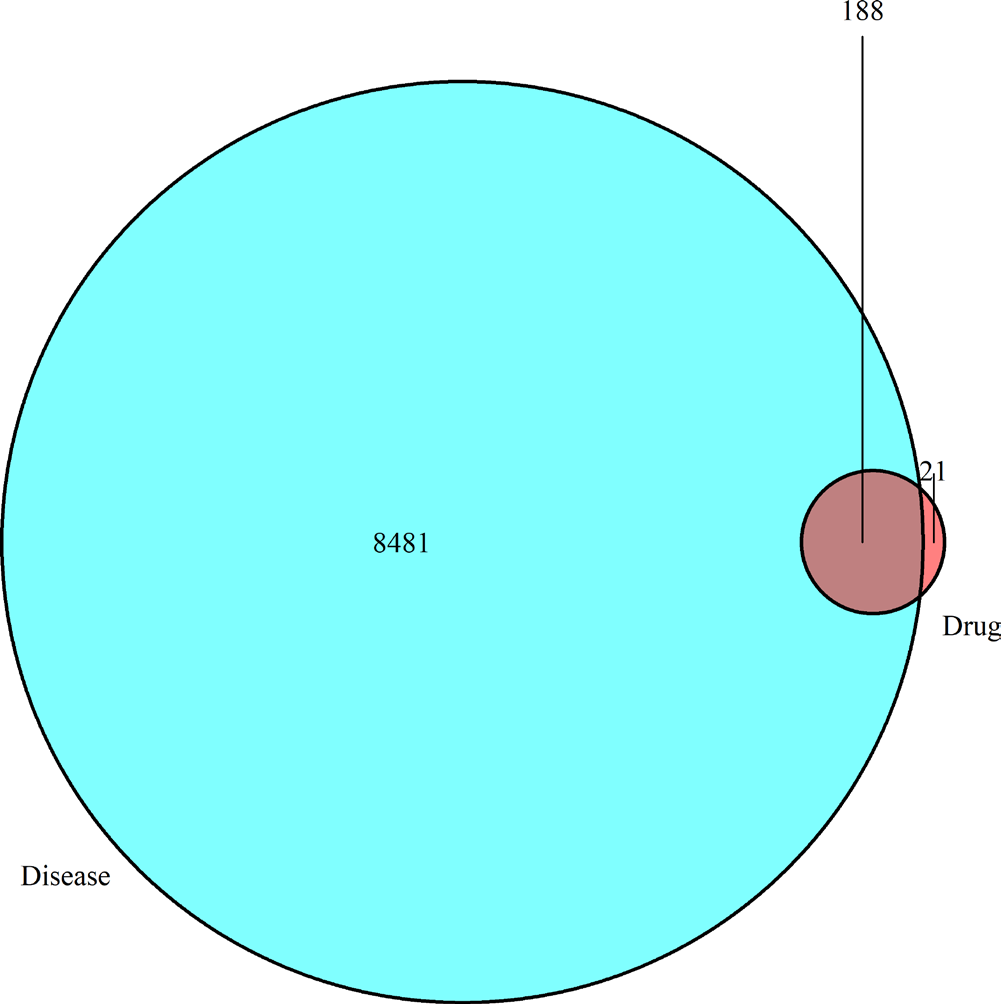
Venn diagram of overlapping target genes between Taohes Chengqi Decoction and constipation.

In parallel, 8481 constipation-associated targets were collected from the GeneCards and OMIM databases. Intersection analysis revealed 188 common genes shared between Taohes Chengqi Decoction and constipation (Fig. 1), suggesting potential therapeutic targets through which Taohes Chengqi Decoction may exert its pharmacological effects.

### 3.2. Construction of the PPI network of shared targets

**Figure 2.**
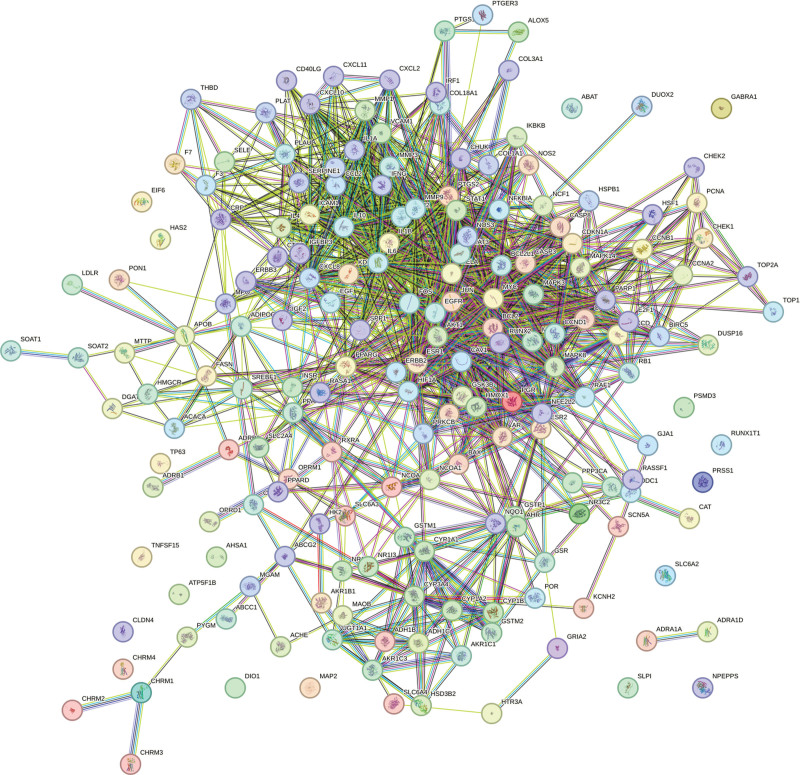
Protein–protein interaction (PPI) network of shared targets between Taohes Chengqi Decoction and constipation.

The 188 overlapping targets of Taohes Chengqi Decoction and constipation were imported into the STRING database under the “Multiple proteins” setting, with the minimum interaction score set at the threshold of 0.700. Isolated nodes were retained, and the drug–disease PPI network was constructed (Fig. 2). The clustering coefficient was 0.616, and the network density was 0.211. The PPI network was divided into 7 distinct modules.

Topological analysis of the network was performed in Cytoscape, ranking nodes by degree value to identify hub proteins. The results indicated that AKT1 (protein kinase B, degree = 258), IL6 (interleukin-6, degree = 238), IL1B (IL1β, degree = 224), and JUN (AP-1 transcription factor subunit, degree = 222) occupied central positions within the network, suggesting pivotal roles in overall regulation. Additional proteins, including CASP3 (caspase-3), PTGS2 (cyclooxygenase-2), STAT3 (signal transducer and activator of transcription 3), hypoxia-inducible factor-1α, estrogen receptor 1, and EGFR (epidermal growth factor receptor) (degree = 200–214), also displayed high connectivity, highlighting their importance in processes such as signal transduction, inflammatory response, apoptosis, and transcriptional regulation.

**Figure 3.**
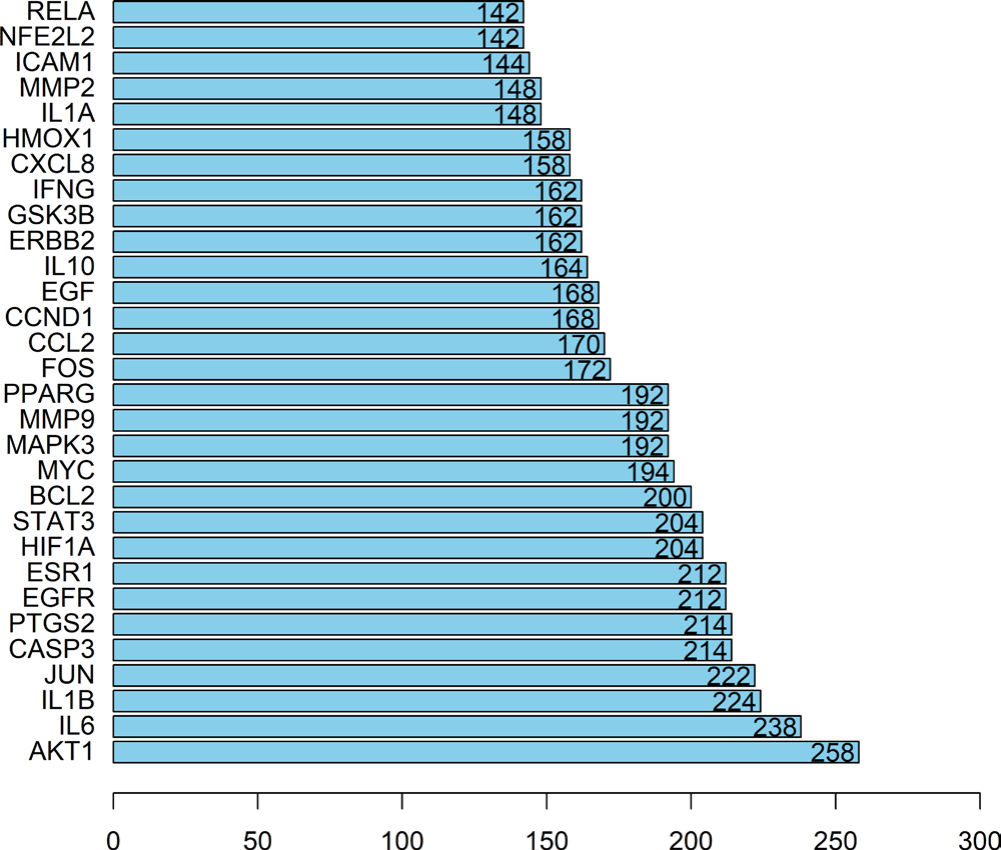
Bar plot of key hub proteins in the PPI network. PPI = protein–protein interaction.

In contrast, proteins such as RELA (NF-κB p65 subunit), nuclear factor erythroid 2-related factor 2, intercellular adhesion molecule 1, and matrix metalloproteinase-2 (degree = 142–148) exhibited relatively lower connectivity, but may nonetheless participate in specific regulatory events (Fig. 3).

### 3.3. Construction of the drug–target–disease network

**Figure 4.**
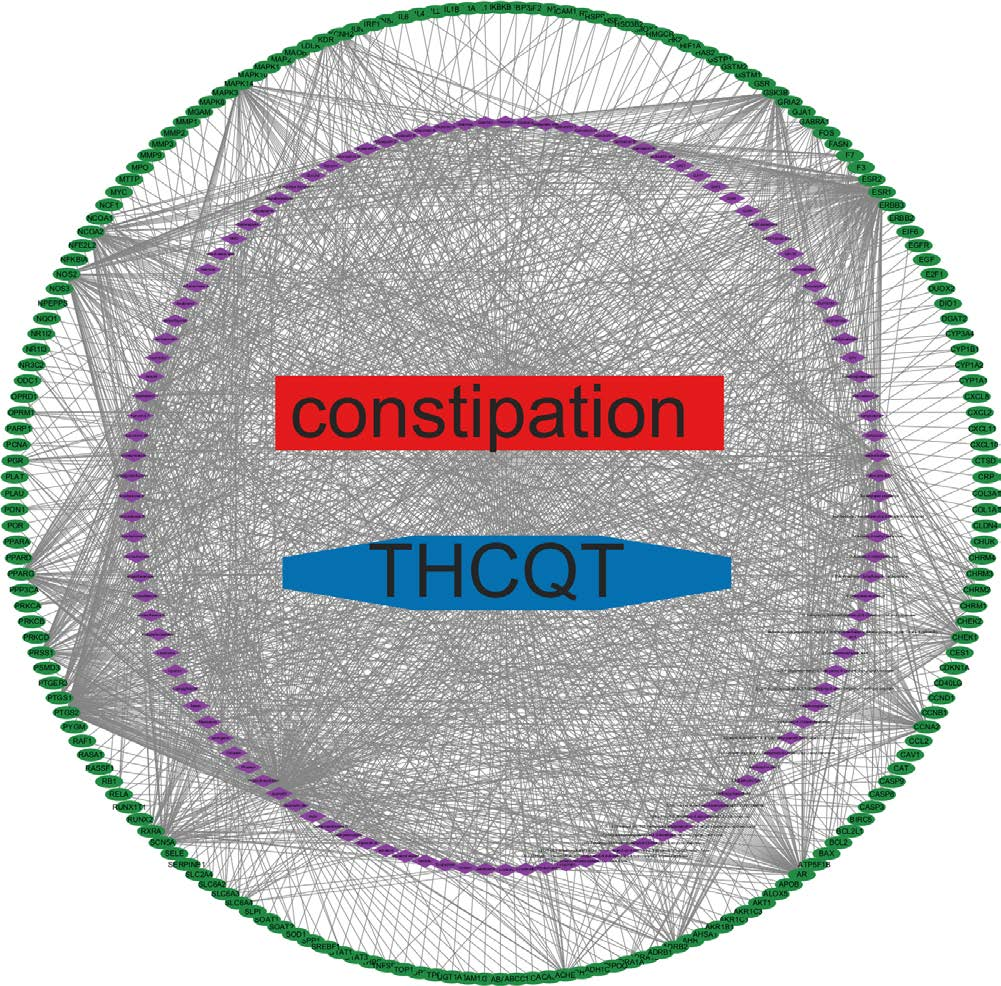
Drug–target–disease interaction network of Taohes Chengqi Decoction in constipation (note: THCQT refers to Taohes Chengqi Decoction).

By integrating the potential targets of Taohes Chengqi Decoction with constipation-related targets, a comprehensive “drug–target–disease” interaction network was established (Fig. 4). In this network, red nodes represent the disease (constipation), blue nodes represent Taohes Chengqi Decoction, green nodes denote constipation-related protein targets, and purple nodes indicate drug-associated targets. The quantitative network parameters were calculated using the NetworkAnalyzer tool in Cytoscape 3.10.0. The drug–target–disease network comprised 186 nodes and 7276 edges, with an average number of neighbors of 39.118. The characteristic path length was 1.901, and the clustering coefficient was 0.616, indicating a high degree of local interconnectivity among nodes. The network density was 0.211, suggesting a moderately compact structure. The network contained a single connected component with no self-loops, and 3838 multi-edge node pairs were identified. These parameters collectively demonstrate that the network exhibits strong connectivity and cohesive topological properties, consistent with the multi-target regulatory features of traditional Chinese medicine formulations.

The overall topological structure revealed a pronounced overlap between Taohes Chengqi Decoction and constipation at the target level, with extensive shared proteins forming a densely interconnected network. Further analysis highlighted several hub nodes occupying central positions, including AKT1 (protein kinase B), IL6, IL1β, STAT3, CASP3, and EGFR. The high connectivity of these proteins suggests that they may play pivotal roles in mediating the therapeutic effects of Taohes Chengqi Decoction against constipation.

### 3.4. GO enrichment analysis

GO enrichment analysis of the overlapping targets between Taohes Chengqi Decoction and constipation revealed significant enrichment in multiple molecular functions. The most prominent categories included DNA-binding transcription factor binding, RNA polymerase II-specific DNA-binding transcription factor binding, ligand-activated transcription factor activity, and nuclear receptor activity, all of which exhibited high enrichment scores and strong statistical significance.

**Figure 5.**
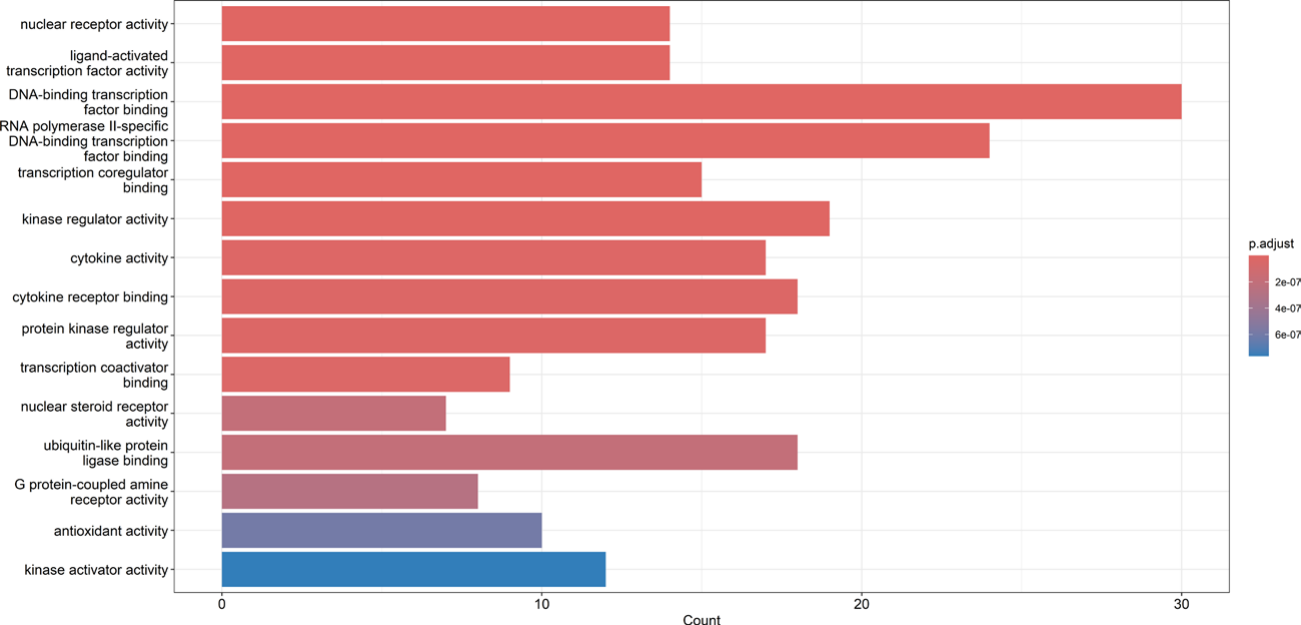
Bar plot of gene ontology (GO) enrichment analysis of overlapping targets.

In addition, notable enrichment was observed in cytokine activity, cytokine receptor binding, protein kinase regulator activity, ubiquitin-like protein ligase binding, and kinase regulator/activator activity (Fig. 5). These findings suggest that Taohes Chengqi Decoction may exert therapeutic effects through the modulation of transcriptional regulation, receptor-mediated signaling, and kinase activity.

### 3.5. KEGG pathway enrichment analysis and key pathway exploration

**Figure 6.**
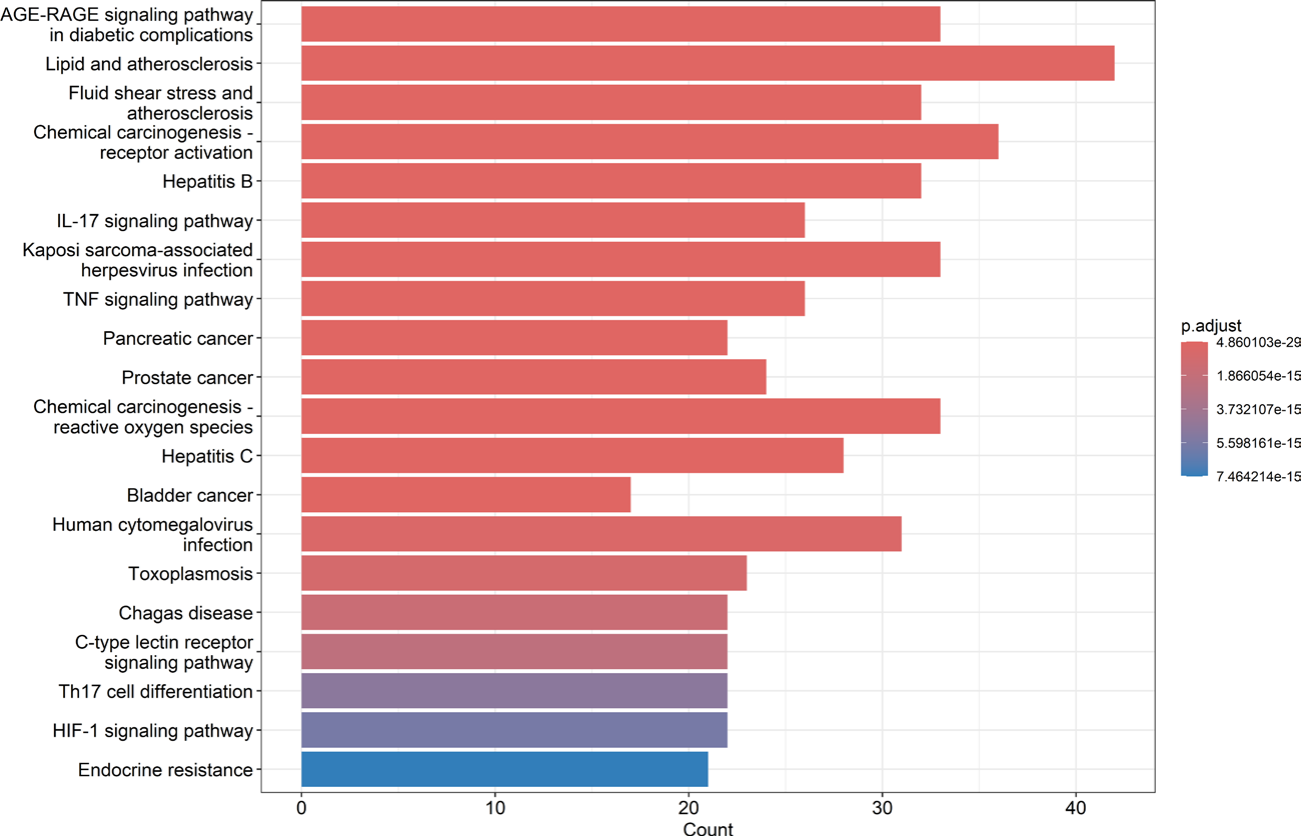
Bar plot of KEGG pathway enrichment analysis of overlapping targets. KEGG = Kyoto encyclopedia of genes and genomes.

KEGG pathway enrichment analysis of overlapping targets between Taohes Chengqi Decoction and constipation revealed significant enrichment across multiple signaling pathways (Fig. 6). Prominent metabolic and stress-related pathways included the advanced glycation end-product binding to their receptor (AGE–RAGE) signaling pathway in diabetic complications, lipid and atherosclerosis, fluid shear stress and atherosclerosis, and chemical carcinogenesis (receptor activation and reactive oxygen species-related).

In parallel, inflammatory and immune-related pathways such as the IL-17 signaling pathway, TNF signaling pathway, HIF-1 signaling pathway, and C-type lectin receptor signaling pathway were markedly enriched. Additional enrichment was observed in tumor- and infection-related pathways, including pancreatic cancer, prostate cancer, bladder cancer, hepatitis B, and human cytomegalovirus infection. Collectively, these findings suggest that Taohes Chengqi Decoction-constipation targets are primarily involved in inflammation, immune regulation, metabolic dysregulation, and tumor-related pathways.

**Figure 7.**
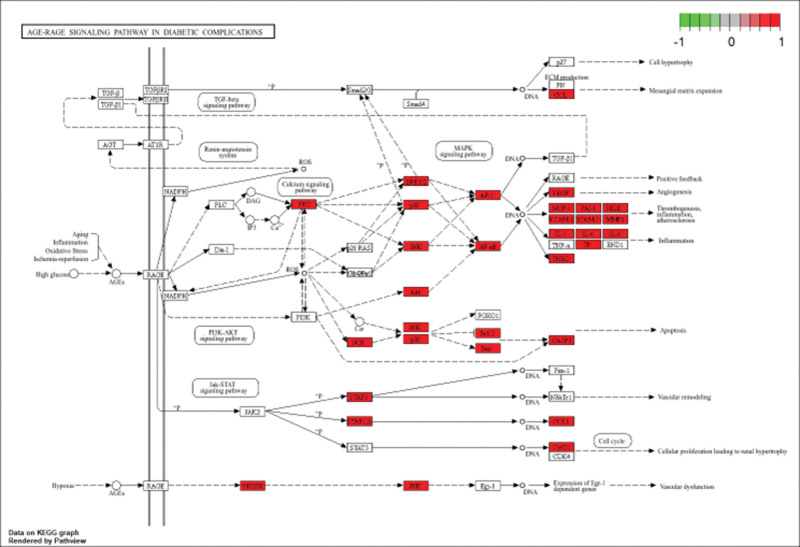
AGE–RAGE signaling pathway. Note: genes/proteins marked in red represent targets significantly enriched or upregulated in this study. The pathway is primarily activated through AGE–RAGE interactions, triggering downstream signaling cascades including MAPK, PI3K–Akt, and JAK–STAT, thereby contributing to inflammatory responses, oxidative stress, apoptosis, and vascular dysfunction. AGE–RAGE = advanced glycation end-product binding to their receptor, STAT3 = signal transducer and activator of transcription 3.

Among the enriched pathways, the AGE–RAGE signaling pathway ranked highest. Pathway mapping revealed significant enrichment of key signaling molecules such as JNK, NF-κB, IL-6, TNF-α, VEGF, and CASP3, indicating a pivotal role of this pathway (Fig. 7). The AGE–RAGE axis is known to mediate biological processes including inflammatory response, oxidative stress, apoptosis, and endothelial dysfunction, all of which are closely associated with the inflammatory microenvironment and tissue injury. These results suggest that the therapeutic effects of Taohes Chengqi Decoction against constipation may be closely linked to modulation of the AGE–RAGE signaling pathway.

## 4. Discussion

TCM has long been recognized for its potential advantages in managing functional disorders,^[13,14]^ and the clinical efficacy of *Taohe Chengqi Decoction* in constipation suggests possible therapeutic benefits. However, unlike single-compound drugs, TCM formulas comprise multiple herbs and diverse bioactive constituents, posing challenges for the elucidation of their precise mechanisms. To address this complexity, we applied a network pharmacology approach to systematically predict the potential mechanisms through which Taohe Chengqi Decoction may exert its therapeutic effects against constipation. By integrating the PPI network, drug–target–disease network, and KEGG pathway enrichment analyses, this study proposes a multi-target and multi-pathway framework underlying the potential pharmacological actions of Taohe Chengqi Decoction.

In the PPI network, AKT1, IL6, IL1B, and JUN exhibited the highest degree values and occupied central positions, suggesting that these proteins may serve as important nodes in the overall regulatory network. AKT1^[15]^ has been widely reported as a key regulator of cell growth, proliferation, and apoptosis, while IL6 and IL1B are well-known mediators of inflammatory cascades that may contribute to intestinal dysfunction. It should be noted, however, that there is currently limited direct evidence linking IL6 and IL1B specifically to constipation-related inflammation, and their potential roles warrant further investigation. JUN, as a major component of the AP-1 transcription factor complex, is involved in transcriptional regulation and stress responses. Collectively, these findings suggest that inflammation, apoptosis, and aberrant signaling pathways may play integral roles in constipation pathophysiology. In addition, CASP3, PTGS2, STAT3, hypoxia-inducible factor-1α, estrogen receptor 1, and EGFR showed high connectivity, indicating that these molecules may be involved in processes such as apoptosis, inflammatory mediator synthesis, transcriptional regulation, and endocrine modulation. These results support the hypothesis that Taohe Chengqi Decoction may potentially modulate multiple targets to achieve therapeutic effects.

The drug–target–disease network revealed a highly interconnected and systemic relationship between Taohe Chengqi Decoction and constipation, mediated by numerous shared targets. This finding suggests that TCM formulas may not act through a single compound–single target paradigm, but rather through multi-component, multi-target, and multi-pathway interactions. Compared with single-target drugs, Taohe Chengqi Decoction may potentially influence inflammatory cytokines, apoptotic signaling, oxidative balance, and neuro-intestinal regulation, thereby contributing to the restoration of intestinal motility. This network-based pharmacological framework reflects the holistic and integrative principles of TCM theory.

Further KEGG enrichment analysis predicted that the AGE–RAGE signaling pathway^[16,17]^ in diabetic complications was among the most significantly enriched, involving key targets such as JNK, NF-κB, IL6, TNF-α, VEGF, and CASP3. This pathway plays important roles in inflammation, oxidative stress, apoptosis, and endothelial dysfunction (biological processes that may contribute to chronic intestinal injury). Previous studies have suggested that overactivation of AGE–RAGE signaling could promote smooth muscle apoptosis and collagen deposition, potentially impairing intestinal motility. Based on our findings, Taohe Chengqi Decoction may potentially alleviate constipation by modulating or suppressing the excessive activation of the AGE–RAGE pathway, thereby reducing inflammation and oxidative stress and preserving tissue function. Mechanistically, the AGE–RAGE signaling pathway provides a plausible explanation for the potential therapeutic effects of Taohe Chengqi Decoction in constipation. AGEs and their receptor RAGE are known to trigger oxidative stress and activate downstream inflammatory cascades, including NF-κB and MAPK pathways, leading to the release of proinflammatory cytokines such as IL6, TNF-α, and IL1B. Chronic activation of the AGE–RAGE axis has been implicated in tissue fibrosis, neuronal dysfunction, and smooth muscle injury (pathophysiological changes that can impair intestinal motility). In this context, several bioactive compounds identified in Taohe Chengqi Decoction, such as quercetin and kaempferol, have been reported to attenuate AGE–RAGE-mediated oxidative stress and inflammatory responses. Therefore, the enrichment of the AGE–RAGE pathway in our network pharmacology analysis provides a mechanistic rationale suggesting that Taohe Chengqi Decoction may potentially alleviate constipation by mitigating oxidative and inflammatory damage in intestinal tissues.

A related network pharmacology study by Hongbao Liang^[18]^ on *Shouhui Tongbian Capsules* also identified the IL-17 signaling pathway, the AGE–RAGE signaling pathway in diabetic complications, and the thyroid hormone signaling pathway as potential mechanisms in constipation treatment. Notably, 2 of these pathways (IL-17 and AGE–RAGE) were also among the top 20 KEGG-enriched pathways in our analysis of Taohe Chengqi Decoction, further supporting the plausibility of these predicted mechanisms.

Taken together, this study predicts that Taohe Chengqi Decoction may act through multi-target and multi-pathway regulatory mechanisms. The key processes are hypothesized to involve modulation of inflammatory mediators, regulation of apoptosis, restoration of oxidative balance, and improvement of vascular function. These findings provide testable hypotheses for future mechanistic studies and offer a theoretical foundation for understanding the potential molecular basis of Taohe Chengqi Decoction in constipation.

Despite providing a systems-level prediction of the potential mechanisms of Taohe Chengqi Decoction in constipation, several limitations should be acknowledged.

First, the current network pharmacology analysis is based primarily on publicly available databases and computational predictions. As such, the accuracy of target identification depends heavily on the completeness and reliability of these databases, and the predictive power of compound–target associations remains uncertain.

Second, although the KEGG enrichment analysis highlighted several inflammation- and apoptosis-related pathways, no exercise- or neuroregulation-related signaling pathways were detected. This absence may be due to database bias or incomplete annotation of intestinal motility-related targets, and future experimental studies are required to determine whether such mechanisms are involved in Taohe Chengqi Decoction’s therapeutic effects.

Third, while our findings suggest that Taohe Chengqi Decoction may potentially modulate inflammation, oxidative stress, and apoptosis, comprehensive experimental validation (including molecular, cellular, and in vivo assays) is essential to verify these predicted interactions and confirm the functional relevance of the identified hub targets and pathways.

Finally, although Taohe Chengqi Decoction is a representative traditional Chinese medicine formula, its mechanistic and therapeutic profile should be compared with conventional treatments for constipation, such as osmotic or stimulant laxatives and prokinetic agents. Such comparative analyses will help clarify whether Taohe Chengqi Decoction acts through complementary or distinct molecular mechanisms, and whether it offers potential advantages in modulating systemic inflammatory or oxidative pathways.

## 5. Conclusion

This study systematically explored and predicted the potential mechanisms of Taohe Chengqi Decoction in the treatment of constipation using a network pharmacology approach. The PPI network analysis suggested that AKT1, IL6, IL1B, and JUN may serve as central hub genes, potentially involved in inflammatory responses, apoptosis, and signaling dysregulation. The drug–target–disease network indicated extensive overlap between Taohe Chengqi Decoction-related and constipation-associated targets, suggesting the multi-component, multi-target, and multi-pathway nature of traditional Chinese medicine formulations. KEGG enrichment analysis predicted the involvement of the AGE–RAGE signaling pathway and related biological processes (such as inflammation, apoptosis, and oxidative stress) as possible mechanisms underlying Taohe Chengqi Decoction’s therapeutic potential.

In summary, Taohe Chengqi Decoction may potentially act against constipation through coordinated modulation of multiple signaling pathways, potentially influencing inflammatory mediators, apoptosis, and oxidative stress to improve intestinal function. These results offer a theoretical basis and hypothesis for future mechanistic and experimental studies, providing useful insights into the possible molecular basis and clinical relevance of Taohe Chengqi Decoction.

Supplemental Digital Contents “GO.xlsx, KEGG.xlsx” are available for this article (https://links.lww.com/MD/R306; https://links.lww.com/MD/R307).

### Acknowledgments

Thanks to Mr. Chen Xu for his outstanding contribution to this article.

## Author contributions

**Methodology:** Yulai Yin.

**Software:** Yulai Yin, Chen Xu.

**Validation:** Yulai Yin, Yixuan Xie, Shufa Tan, Chen Xu.

**Visualization:** Yulai Yin, Shufa Tan, Yinxu Zhang, Chen Xu.

**Writing – original draft:** Yulai Yin, Yixuan Xie, Shufa Tan.

**Writing – review & editing:** Yinxu Zhang, Chen Xu.

## Supplementary Material





## References

[1] Barberio B Judge C Savarino EV Ford AC. Global prevalence of functional constipation according to the Rome criteria: a systematic review and meta-analysis. Lancet Gastroenterol Hepatol. 2021;6:638–48. 34090581 10.1016/S2468-1253(21)00111-4

[2] Scott SM Simrén M Farmer AD. Chronic constipation in adults: contemporary perspectives and clinical challenges. 1: epidemiology, diagnosis, clinical associations, pathophysiology and investigation. Neurogastroenterol Motil. 2021;33:e14050. 33263938 10.1111/nmo.14050

[3] O’Donnell MT Haviland SM. Functional constipation and obstructed defecation. Surg Clin North Am. 2024;104:565–78. 38677821 10.1016/j.suc.2023.11.007

[4] Tang JL Liu BY Ma KW. Traditional Chinese medicine. Lancet. 2008;372:1938–40. 18930523 10.1016/S0140-6736(08)61354-9

[5] Zhang XM. The evidential research on the versions of Shanghan Lun Ben Zhi. Zhonghua Yi Shi Za Zhi. 2019;49:29–33. 30970422 10.3760/cma.j.issn.0255-7053.2019.01.006

[6] Li DY Dai YK Zhang YZ. Systematic review and meta-analysis of traditional Chinese medicine in the treatment of constipation-predominant irritable bowel syndrome. PLoS One. 2017;12:e0189491. 29253850 10.1371/journal.pone.0189491 PMC5734785

[7] Gao CC Li GW Wang TT. Rhubarb extract relieves constipation by stimulating mucus production in the colon and altering the intestinal flora. Biomed Pharmacother. 2021;138:111479. 33774313 10.1016/j.biopha.2021.111479

[8] Ru J Li P Wang J. TCMSP: a database of systems pharmacology for drug discovery from herbal medicines. J Cheminform. 2014;6:13. 24735618 10.1186/1758-2946-6-13 PMC4001360

[9] Wang Y Xiao J Suzek TO. PubChem’s BioAssay Database. Nucleic Acids Res. 2012;40:D400–412. 22140110 10.1093/nar/gkr1132 PMC3245056

[10] Pundir S Martin MJ O’Donovan C. UniProt protein knowledgebase. Methods Mol Biol. 2017;1558:41–55. 28150232 10.1007/978-1-4939-6783-4_2 PMC5565770

[11] Stelzer G Rosen N Plaschkes I. The genecards suite: from gene data mining to disease genome sequence analyses. Curr Protoc Bioinformatics. 2016;54:1.30.31–31.30.33. 10.1002/cpbi.5 27322403

[12] Amberger JS Hamosh A. Searching online Mendelian inheritance in man (OMIM): a knowledgebase of human genes and genetic phenotypes. Curr Protoc Bioinformatics. 2017;58:1.2.1–1.2.12. 10.1002/cpbi.27 PMC5662200 28654725

[13] Wang K Qiu H Chen F Cai P Qi F. Considering traditional Chinese medicine as adjunct therapy in the management of chronic constipation by regulating intestinal flora. Biosci Trends. 2024;18:127–40. 38522913 10.5582/bst.2024.01036

[14] Wang L Wu F Hong Y Shen L Zhao L Lin X. Research progress in the treatment of slow transit constipation by traditional Chinese medicine. J Ethnopharmacol. 2022;290:115075. 35134487 10.1016/j.jep.2022.115075

[15] Kang J Yang X Sui N. Therapeutic mechanism of Zhishi decoction regulating P38/MAPK signaling pathway on functional constipation (FC). Comb Chem High Throughput Screen. 2025;28:3170–87. 39917918 10.2174/0113862073332162241126105559

[16] Wang M Ma X Gao C. Rutin attenuates inflammation by downregulating AGE–RAGE signaling pathway in psoriasis: network pharmacology analysis and experimental evidence. Int Immunopharmacol. 2023;125:111033. 38149569 10.1016/j.intimp.2023.111033

[17] Zhang N Chen P Liang X. Luteolin targets the AGE–RAGE signaling to mitigate inflammation and ferroptosis in chronic atrophic gastritis. Aging (Albany NY). 2024;16:10918–30. 38917486 10.18632/aging.205969 PMC11272119

[18] Liang HB Li R Yao JC Qin GF Zhang H Zhang GM. Mechanism of Shouhui Tongbian Capsules in treating constipation based on network pharmacology and molecular docking. Zhongguo Zhong Yao Za Zhi. 2021;46:511–9. 33645014 10.19540/j.cnki.cjcmm.20201117.406

